# Emergence of Novel Reassortant H1N1 Avian Influenza Viruses in Korean Wild Ducks in 2018 and 2019

**DOI:** 10.3390/v13010030

**Published:** 2020-12-26

**Authors:** Thuy-Tien Thi Trinh, Bao Tuan Duong, Anh Thi Viet Nguyen, Hien Thi Tuong, Vui Thi Hoang, Duong Duc Than, SunJeong Nam, Haan Woo Sung, Ki-Jung Yun, Seon-Ju Yeo, Hyun Park

**Affiliations:** 1 Zoonosis Research Center, Department of Infection Biology, School of Medicine, Wonkwang University, Iksan 570-749, Korea; trinhthithuytien.k56@hus.edu.vn (T.-T.T.T.); bao2dt@gmail.com (B.T.D.); nguyenthivietanh.k56@hus.edu.vn (A.T.V.N.); tuonghien23@gmail.com (H.T.T.); hoangvui169@gmail.com (V.T.H.); ducduong27189@gmail.com (D.D.T.); 2 Division of EcoScience, Ewha University, Seoul 03760, Korea; sjnam01@daum.net; 3 College of Veterinary Medicine, Kangwon National University, Chuncheon 200-701, Korea; sunghw@kangwon.ac.kr; 4 Department of Pathology, School of Medicine, Wonkwang University, Iksan 570-749, Korea; kjyun@wku.ac.kr; 5 Department of Tropical Medicine and Parasitology, College of Medicine, Seoul National University, Seoul 03080, Korea

**Keywords:** avian influenza, H1N1, isolates, mammal adaptation, pathogenesis

## Abstract

Influenza A virus subtype H1N1 has caused global pandemics like the “Spanish flu” in 1918 and the 2009 H1N1 pandemic several times. H1N1 remains in circulation and survives in multiple animal sources, including wild birds. Surveillance during the winter of 2018–2019 in Korea revealed two H1N1 isolates in samples collected from wild bird feces: KNU18-64 (A/*Greater white-fronted goose*/South Korea/KNU18-64/2018(H1N1)) and WKU19-4 (A/wild bird/South Korea/WKU19-4/2019(H1N1)). Phylogenetic analysis indicated that M gene of KNU18-64(H1N1) isolate resembles that of the Alaskan avian influenza virus, whereas WKU19-4(H1N1) appears to be closer to the Mongolian virus. Molecular characterization revealed that they harbor the amino acid sequence PSIQRS↓GLF and are low-pathogenicity influenza viruses. In particular, the two isolates harbored three different mutation sites, indicating that they have different virulence characteristics. The mutations in the PB1-F2 and PA protein of WKU19-4(H1N1) indicate increasing polymerase activity. These results corroborate the kinetic growth data for WKU19-4 in MDCK cells: a dramatic increase in the viral titer after 12 h post-inoculation compared with that in the control group H1N1 (CA/04/09(pdm09)). The KNU18-64(H1N1) isolate carries mutations indicating an increase in mammal adaptation; this characterization was confirmed by the animal study in mice. The KNU18-64(H1N1) group showed the presence of viruses in the lungs at days 3 and 6 post-infection, with titers of 2.71 ± 0.16 and 3.71 ± 0.25 log10(TCID50/mL), respectively, whereas the virus was only detected in the WKU19-4(H1N1) group at day 6 post-infection, with a lower titer of 2.75 ± 0.51 log10(TCID50/mL). The present study supports the theory that there is a relationship between Korea and America with regard to reassortment to produce novel viral strains. Therefore, there is a need for increased surveillance of influenza virus circulation in free-flying and wild land-based birds in Korea, particularly with regard to Alaskan and Asian strains.

## 1. Introduction

Based on 18 hemagglutinin (HA) and 11 neuraminidase (NA) surface proteins, influenza viruses are classified into a wide array of subtypes, some of which are a public health threat [1]. Sixteen HA subtypes (H1–H16) and 9 NA subtypes (N1–N9) are avian influenza viruses (AIVs), whereas subtypes H17N10 and H18N11 have only been detected in bats [2].

H1 AIVs are a type of influenza A virus that can be transmitted from birds to mammals; these viruses can co-circulate among wild birds, pigs, and human beings [3]. Influenza viruses are isolated from human for the first time in 1930s [4]. Since then, the H1N1 subtype virus in particular has caused two seriously deadly pandemics in human beings including the 1918 “Spanish flu” pandemic and the 2009 H1N1 pandemic [5]. The 1918 H1N1 flu pandemic originated from an avian population and, subsequently, infected porcine and human populations at approximately the same time, although it was not pathogenic in birds [6]. In 1979, avian-like H1N1 viruses entered the Eurasian swine population and co-circulated with the classical swine virus in Eurasia [3,7]. The 1957 and 1968 pandemics, which were caused by subtypes H2N2 and H3N2, respectively, replaced the H1N1 virus in the human population until its reappearance in 1977. Since then, the H1N1 virus has been circulating in humans along with the seasonal H3N2 virus [8]. A novel H1N1 virus emerged in 2009 worldwide and was introduced in Korean with a unique gene constellation of avian-, porcine-, and human-origin segments. It caused a pandemic, despite the existing human immunity to seasonal H1N1 [9,10]. The low genetic diversity among the viruses suggests that the introduction into humans was caused by a single event or multiple events involving similar viruses [11].

Historically, several highly pathogenic AIVs (HPAI) have infected poultry in Korea, indicating that Korea’s geographic location facilitates the reassortment of avian influenza [12,13,14].

In Korea alone, HPAI outbreaks have been reported five times since 2003 [13,15,16,17,18]. Especially, H5N8 virus represent the fifth and last HPAI outbreak in January 2014. In addition, H5N8 HPAI viruses were detected from 38 wild birds in 200 poultry farms in Korea in 2014 [19]. Meanwhile, the H5N1 HPAI virus caused the other four outbreaks in 2003, 2006, 2008, and 2010. During these outbreaks, HPAI viruses were transmitted in poultry and live bird markets [20]. Wild birds serve as the reservoir of influenza A virus in nature. While viruses are normally nonpathogenic in wild birds, domestic birds and mammals sometimes suffer significantly morbidity and mortality upon its transmission [21]. Therefore, persistent surveillance of the infectious influenza A subtype (H1N1) virus in birds and swine is important to prevent or control transmission of AIV to human in Korea.

In the current study, we conducted a genetic analysis and a molecular characterization of two isolates of avian H1N1 found in 2018, and we hypothesized the reassortment events that led to the generation of these viruses.

## 2. Materials and Methods

### 2.1. Sample Collection

During the winter season from November 2018 to March 2019, fresh samples of the feces of wild migratory birds were collected from a wild field near riverside in Chuncheon and Kangwa province of Korea. A total of 1800 fresh fecal dropping samples were packed at 2–8 °C and sent to a laboratory within 1 day for further analysis. KNU18-64 (A/*Greater white-fronted goose*/South Korea/KNU18-64/2018(H1N1)) and WKU19-4 (A/wild bird/South Korea/WKU19-4/2019(H1N1)) samples were isolated from the feces of waterfowl obtained from Chuncheon, Korea (37° 55′ 03.12′′, 127° 44′ 15.75′′) on 7 November 2018, and from Kanghwa, Korea (37° 45′ 11.4′′, 126° 30′ 13.3′′) on 17 March 2019 (Figure S1).

### 2.2. Virus Isolation

As described previously [22], the fecal samples were resuspended in phosphate-buffered saline (PBS; pH 7.4), then thoroughly vortexed and centrifuged for 10 min at 3000rpm. The supernatants were collected and filtered using a 0.45-µm membrane (GVS Syringe, Nova-Tech, Kingwood, TX, USA) prior to inoculation into 10-day-old specific-pathogen-free (SPF) chicken embryos (Seng-Jin Inc., Eumsung, Korea). The allantoic fluid was harvested from the embryos after incubation for 3 days at 37 °C in a humidified atmosphere. The presence of the virus was determined by screening for viral HA activity via a hemagglutination assay [23]. The results of the HA activity tests were confirmed by reverse-transcription PCR (RT-PCR) using pan-influenza A MP primers [24].

### 2.3. Viral Genome Extraction and RT-PCR to Confirm the Virus and Identify the Host

To confirm the presence of the virus, the total viral RNA was extracted from the infected allantoic fluid using a Qiagen^®^ Viral RNA Isolation kit (Qiagen, Hilden, Germany) according to the manufacturer’s instructions [25]. The mixed total RNAs were reverse-transcribed and the virus was confirmed the *matrix* gene (MP) coding sequence of influenza A virus amplified by PCR according to the World Health Organization guidelines [24]. Cytochrome c oxidase I (COX1), a 648-bp region of the mitochondrial gene, was amplified from DNA extracted from the fecal samples, and was used as a DNA barcode to identify animal species as described previously [26].

### 2.4. Next-Generation Sequencing (NGS) Using Illumina HiSeq X

NGS was conducted by GnC Bio (Daejeon, Korea) using HiSeq X as previously describled [27]. Briefly, the RNA was evaluated using an Agilent RNA 6000 Pico Kit (Agilent, Santa Clara, CA, USA), and quantified using a BioPhotometer^®^ spectrophotometer (Eppendorf, Hamburg, Germany). The complementary DNA (cDNA) library of the influenza RNA was generated using a QIAseq FX Single Cell RNA Library Kit (Qiagen, Venlo, The Netherlands). The concentration of the cDNA was determined using a LightCycler^®^ qPCR system (Roche, Penzberg, Upper Bavaria, Germany), and the library size was checked using a High Sensitivity D5000 ScreenTape system (Agilent). First, the library was loaded into a flow cell, where fragments were captured on a lawn of surface-bound oligos complementary to the library adapters. Each fragment was amplified into distinct clonal clusters by bridge amplification. When cluster generation was complete, the templates were used for sequencing. Illumina SBS technology utilizes a proprietary reversible terminator-based method that detects single bases as they become incorporated into DNA template strands. Because all four reversible, terminator-bound dNTPs were present during each sequencing cycle, natural competition minimized their incorporation bias and greatly reduced raw error rates, compared to those in other technologies. The method provided highly accurate base-by-base sequencing that virtually eliminated sequence-context-specific errors, even within repetitive sequence regions and homopolymers. The sequencing data were then converted into raw data for analysis [28].

### 2.5. Viral Genomic and Phylogenetic Tree Analyses

Phylogenetic analysis was performed using the MEGA 6.0 ((Molecular Evolutionary Genetics Analysis version 6.0, Pennsylvania State University, PA, USA) software package with the maximum-likelihood method, and the tree topology was evaluated by 1000 bootstrap iterations [29]. In the present study, the closest relative reference virus sequences were found using the online BLAST resource from the National Center For Biotechnology Information (NCBI https://www.ncbi.nlm.nih.gov/) and the Global Initiative on Sharing All Influenza Data (GISAID https://www.gisaid.org/) [22].

### 2.6. Determination of 50% Tissue Culture Infectious Dose (TCID_50_) and 50% Egg Infectious Dose (EID_50_)

To characterize viral pathogenicity, Madin–Darby canine kidney (MDCK) cells obtained from the American Type Culture Collection (ATCC, Manassasa, VA, USA) were used to determine the TCID_50_ titers of the viral isolates via enzyme-linked immunosorbent assay (ELISA), as reported previously [30]. Briefly, a flat-bottomed 96-well cell culture plate was coated with 10^4^ MDCK cells, and incubated at 37 °C in 5% CO_2_ until 90% confluence was reached. Each viral stock was serially diluted ten times and used to inoculate the cells, which were then incubated for 3 days at 37 °C in 5% CO_2_. After incubation, the supernatant was removed by vacuum, washed three times with cold PBS, and fixed with cold acetone. After performing the ELISA, the TCID_50_ titers were calculated using the Reed and Muench method [31]. To evaluate the infectious particles in each viral stock, the EID_50_ titers were determined by serially diluting the viral stocks ten times and using them to inoculate 10-day-old SPF-embryonated chicken eggs. Subsequently, infective amniotic–allantoic fluid (AAF) was harvested on Day 4 after inoculation, and HA activity was used to determine the 50% egg infectious dose [32].

### 2.7. Viral Growth Kinetics in MDCK Cells

The growth kinetics of the following viral strains were evaluated in vitro: A/*Greater white-fronted goose*/South Korea/KNU18-64/2018(H1N1) (abbreviation: KNU18-64(H1N1)); A/wild bird/South Korea/WKU19-4/2019(H1N1) (abbreviation: WKU19-4(H1N1)); and A/California/04-005-MA/2009(H1N1) (abbreviation: CA/04/09(pdm09) (H1N1)). To accomplish this the MDCK cells in the monolayers were infected with the viruses at a multiplicity of infection (MOI) rate of 0.01 plaque-forming units per cell. After incubation for 1 h, the supernatants were removed, washed twice with PBS, and continuously cultured in infected media containing l-1-p-tosylamino-2-phenylethyl chloromethyl ketone-treated trypsin. The viral titers in the culture supernatants were determined at 12, 24, 36, 48, and 72 h post-infection (hpi) via a TCID_50_ assay, as previously described [28].

### 2.8. Animal Study

Six-week-old female BALB/c mice were purchased from Orient Bio (Seongnam, Gyeonggi, Korea). To evaluate the pathogenicity of each isolate in a mammalian host, BALB/c mice were intranasally infected with various EID_50_ viral concentrations (10^4^, 10^5^, or 10^6^ (EID_50_/mouse) (*n* = 5)), and the weight changes and survival rates of the mice were recorded for 15 days post-infection (dpi).

Finally, the BALB/c mice were intranasal challenged with each viral isolate at a concentration of 10^5^ EID_50_ (*n* = 5) (i.e., KNU18-64, WKU19-4, and a control group infected with A/California/04/2009 (CA/04/09)(pdm09)), and the weights and survival rates of the mice were recorded for 15 dpi. To evaluate virus shedding in the lung, at 3, 6, and 15 dpi, the lungs of three mice were collected and weighed, and the viral titer in each lung was determined by TCID_50_ assay. To examine the histopathology of the infected lungs, at 3, 6, and 15 dpi, the lungs from three mice were placed in 10% formalin/saline. Lung tissues in 10% formalin/PBS were processed and embedded in paraffin, and 4–5 μm sections were cut from the paraffin-embedded lung tissues, and mounted on glass slides. An histological examination of the lung sections was carried out by standard hematoxylin and eosin (H&E) staining and light microscopy (magnification × 100). This study was approved by the Animal Ethics Committee of the Wonkwang University (WKU19-64), December 19, 2019 and all methods were carried out in accordance with relevant guidelines and regulations.

### 2.9. Statistical Analysis

All the data were statistically analyzed using GraphPad Prism version 5.0 (GraphPad Software, La Jolla, CA, USA). Two-way analysis of variance (ANOVA) was used to assess the viral replication kinetics in the MDCK cells, and one-way ANOVA was used to compare the weights of the virus-infected mouse lungs.

## 3. Results

### 3.1. Genome Characterization of Two H1N1 Isolates

The isolate genome sequences were then deposited at GenBank (accession numbers MN584878 to MN584885 for the KNU18-64 isolate, and MT821115 to MT821122 for WKU19-4). The GenBank accession numbers of the eight gene segments and the highest nucleotide identities from the GenBank database are shown in Table 1, with sequence identities of 98.07 to 99.64% with regard to the H1N1 isolates investigated in the present study.

**Figure 1:**
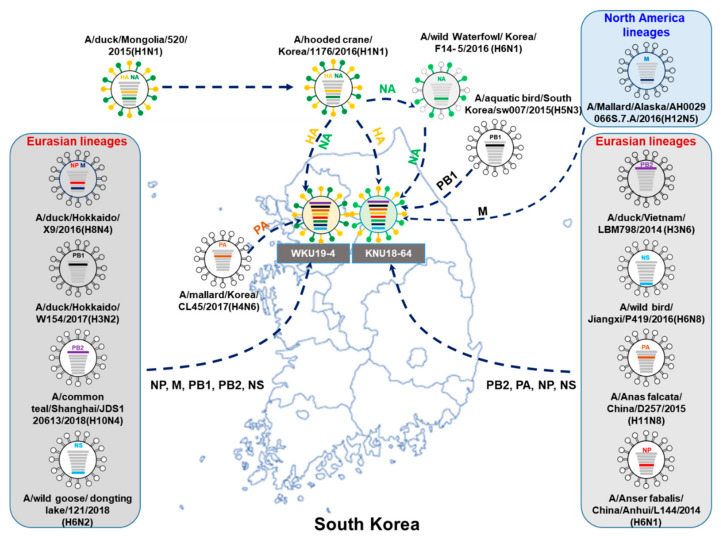
Putative origins of the genes comprising the KNU18-64 (A/*Greater white-fronted goose*/South Korea/KNU18-64/2018(H1N1)) and WKU19-4 (A/wild bird/South Korea/WKU19-4/2019(H1N1)) strains.

The putative gene segments of the KNU18-64 and WKU19-4 isolates are illustrated in Figure 1, based on their nucleotide identities and the phylogenetic tree.

As described in Figure 1, the HA genes in both KNU18-64 and WKU19-4 most closely resemble the Mongolia avian H1N1 strain (A/duck/Mongolia/520/2015(H1N1).

The KNU18-64(H1N1) isolate from the East Asian wild bird flyway appears to have been reassorted, because the internal genes PA, NP, and NS most closely resemble those of the Chinese wild bird influenza virus, and PB2 is closest to the PB2 gene from the H3N6 isolate found in Vietnamese ducks.

Moreover, the NA and PB1 genes of this isolate are similar to those of the original Korean virus.

The internal MP gene of this KNU18-64 isolate is similar to the corresponding gene in the H12N5 isolate from Alaska.

This implies that these viruses were reassorted during the intersection of the wild bird flyway between the East Asian–Australian Flyway (EAAF) and the West Pacific Flyway.

The WKU2019-4(H1N1) isolate most closely resembles the H1N1 Mongolian isolate, with high similarity between the respective HA and NA genes, and it also has three internal genes (PB1, NP, and MP) that are closest to the original avian influenza virus strains from Hokkaido, Japan (H3N2 and H8N4 from ducks).

Furthermore, this virus also includes three other genes (PB2, PA, and NS) that are similar to those of the influenza virus from Korea and China.

These two new isolated viruses (KNU18-64 and WKU19-4) represent two different reassortments of the H1N1 subtype virus that were present in the migratory bird population during the winter of 2018–2019.

**Figure 2:**
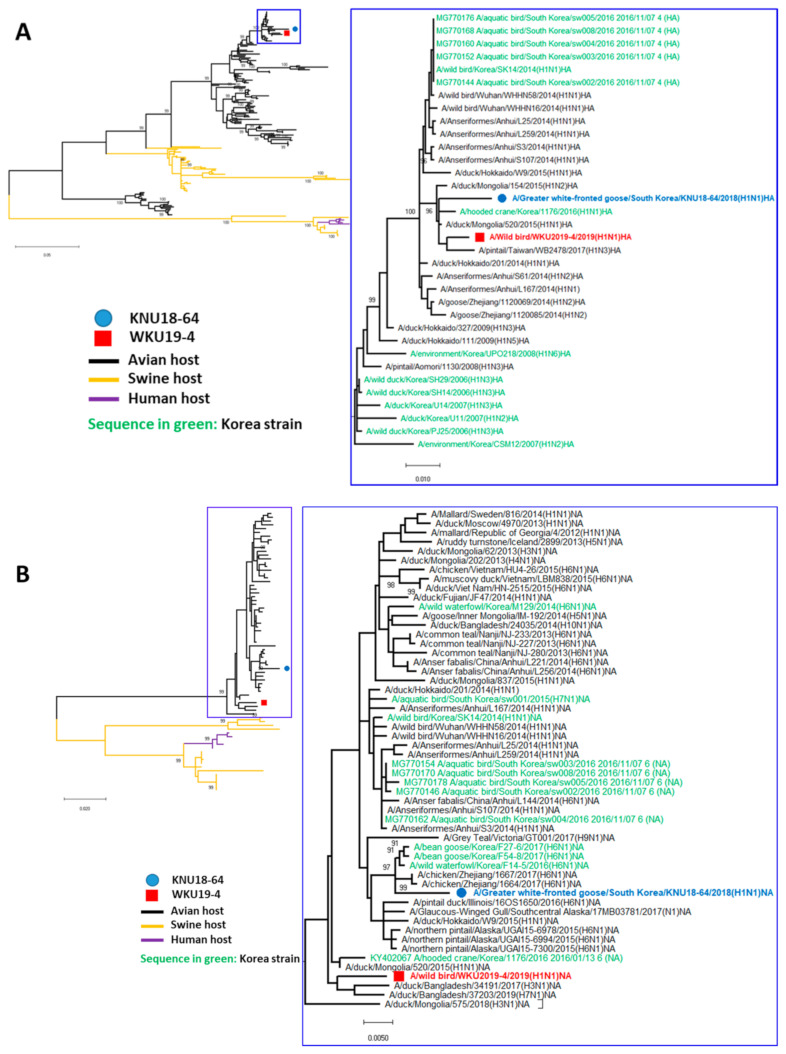

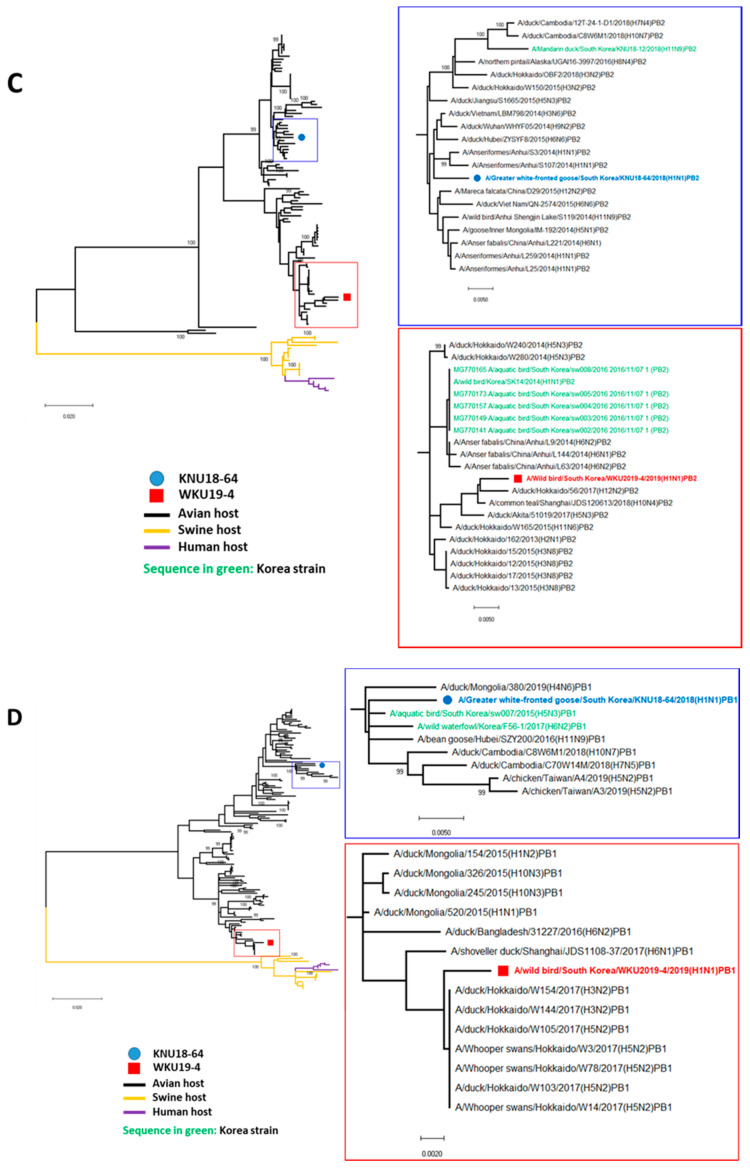

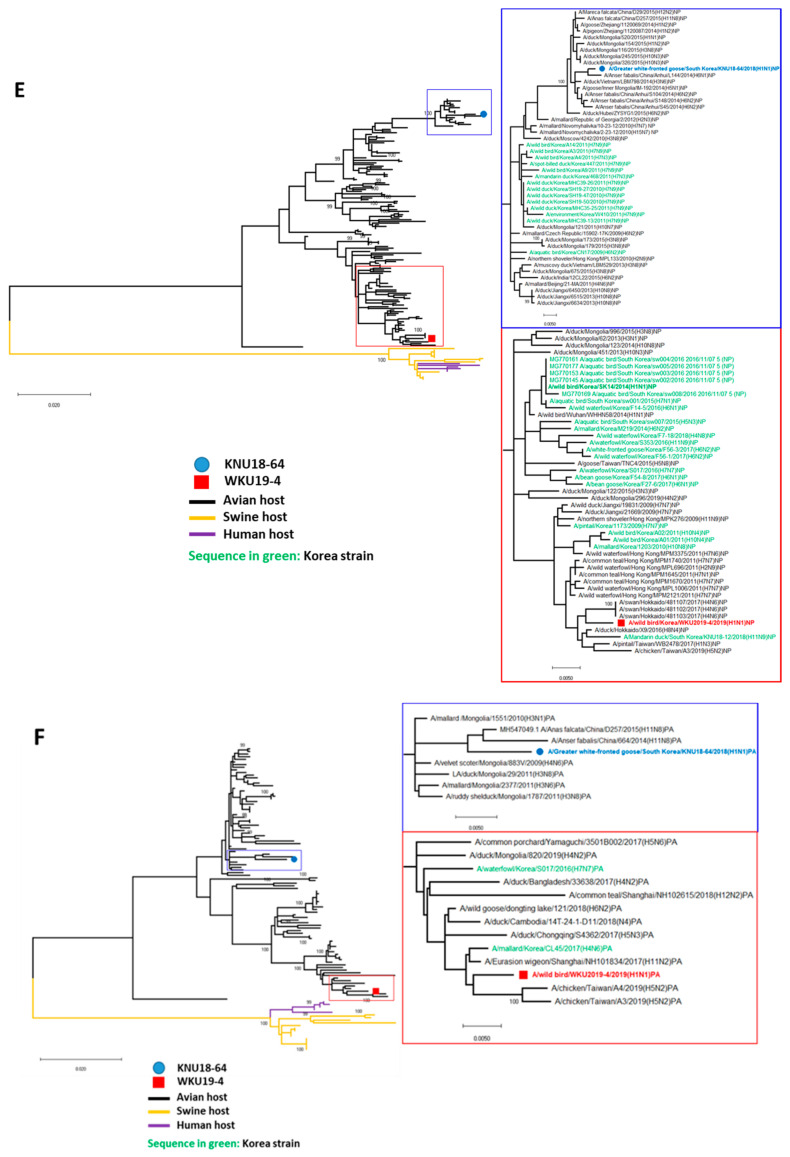

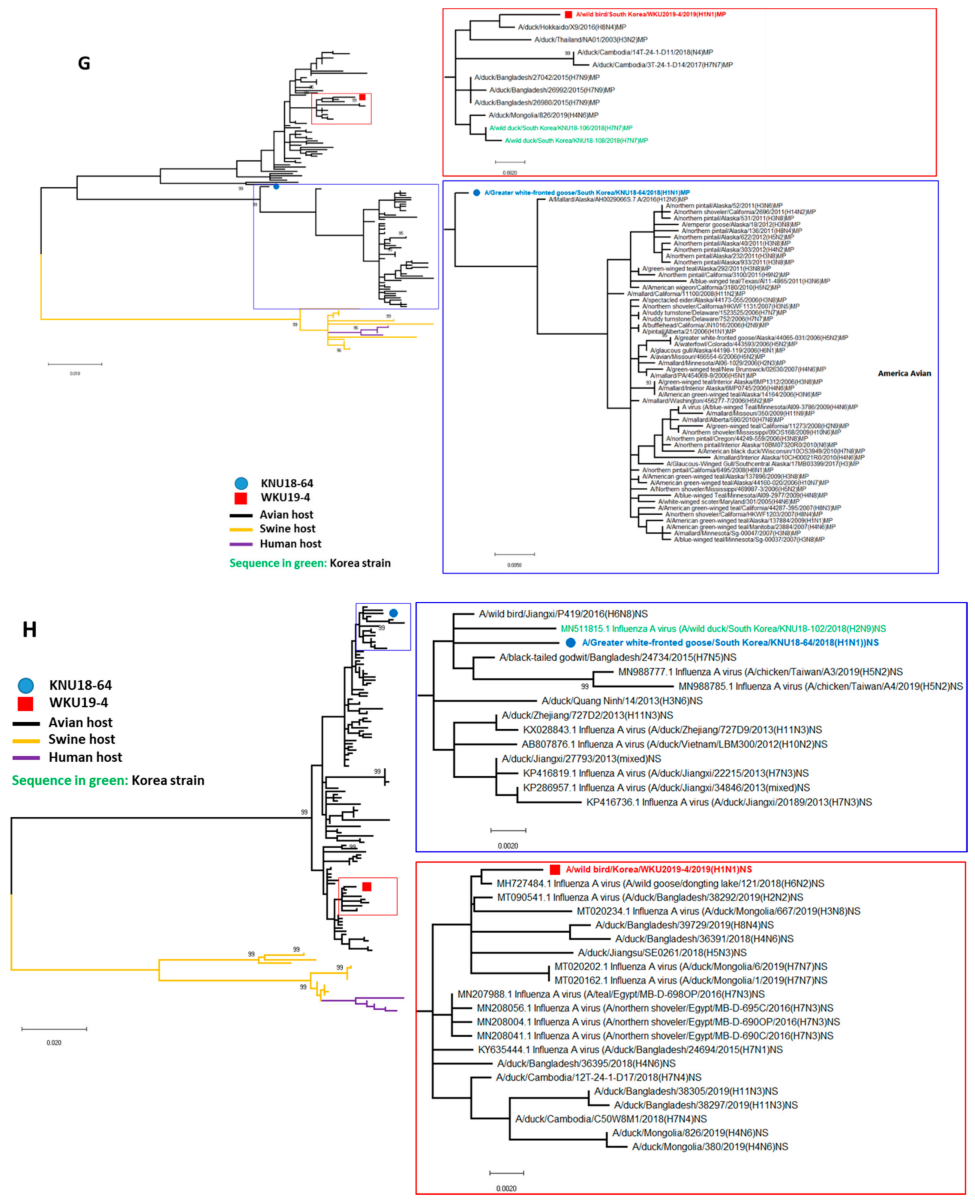
Phylogenetic trees based on the nucleotide sequences of HA (**A**), NA (**B**), PB2 (**C**), PB1 (**D**), NP (**E**), PA (**F**), MP (**G**), and NS (**H**). The trees were generated by the neighbor-joining method using MEGA 6.0 software with bootstrap replication (1000 bootstrap iterations). The KNU18-64 strain (A/*Greater white-fronted goose*/South Korea/KNU18-64/2018(H1N1)) is indicated by blue circles and the WKU19-4 strain (A/wild bird/South Korea/WKU19-4/2019(H1N1)) is indicated by red boxes. The avian, swine, and human hosts are represented in black, orange, and purple, respectively. The Korean strains are shown in green.

We carried out phylogenic analyses of the eight genes from both viral strains to assess their genetic relationships with those of domestic poultry and wild birds in Korea and neighboring countries using data from the NCBI and GISAID with selected sequence with more than 99% and 90% of query cover and identity, respectively. The results revealed that eight genes (PB2, PB1, PA, HA, NP, NA, M, and NS) in our H1N1 strains were distributed in East Asian lineages and a North American avian lineage. The phylogenetic data revealed that the H1N1 influenza strains investigated in the present study are of avian origin, and are phylogenetically distant from the H1N1 swine virus and the human-infecting strain. This substantial divergence from the swine- and human-infecting strains is demonstrated in Figure 2 by the sizable distance between the novel isolates (represented by black branches) and the swine strain (yellow branches) and human-infecting strain (purple branches).

In the present study, the HA genes of KNU18-64 and WKU19-4 were closely related to those of avian H1N1 subtype isolates obtained from Mongolia in 2015, with identities of 98.53% and 99.06%, respectively (Table 1). As with the HA gene, the WKU19-4 NA gene most closely resembles that of the avian H1N1 subtype isolates obtained from Mongolia in 2015, whereas the KNU18-64 NA gene most closely resembles the Korean H6N1 strain obtained in 2016 (Figure 2A,B). The phylogenetic tree and the genomic highest identities demonstrate that the PB1, PB2, NP, and MP genes of the WKU19-4 isolate may have been introduced into Korea by Japanese strains with percentile identities of 98.86%, 99.56%, 99.2%, and 99.49%, respectively. However, the PA and NS genes of this isolate most closely resemble those of the Chinese/Eurasian lineage. Except for the MP gene, which closely resembles that of a North American lineage obtained in 2016, the KNU18-64 isolate internal gene sequences (PB2, PB1, PA, NP, NS) resemble those of the Eurasian/Mongolian/Chinese isolate (Figure 2C–G). This implies that the H1N1 virus detected in Mongolia in 2015 subsequently migrated to Korea and that since 2016, there have been some reassortments with Japanese and Eurasian strains to generate WKU19-4 and KNU18-64.

### 3.2. A/Greater White-Fronted Goose/South Korea/KNU18-64/2018(H1N1) and A/Wild Bird/South Korea/WKU19-4/2019(H1N1) were Generated as a Result of Reassortment Events

**Figure 3:**
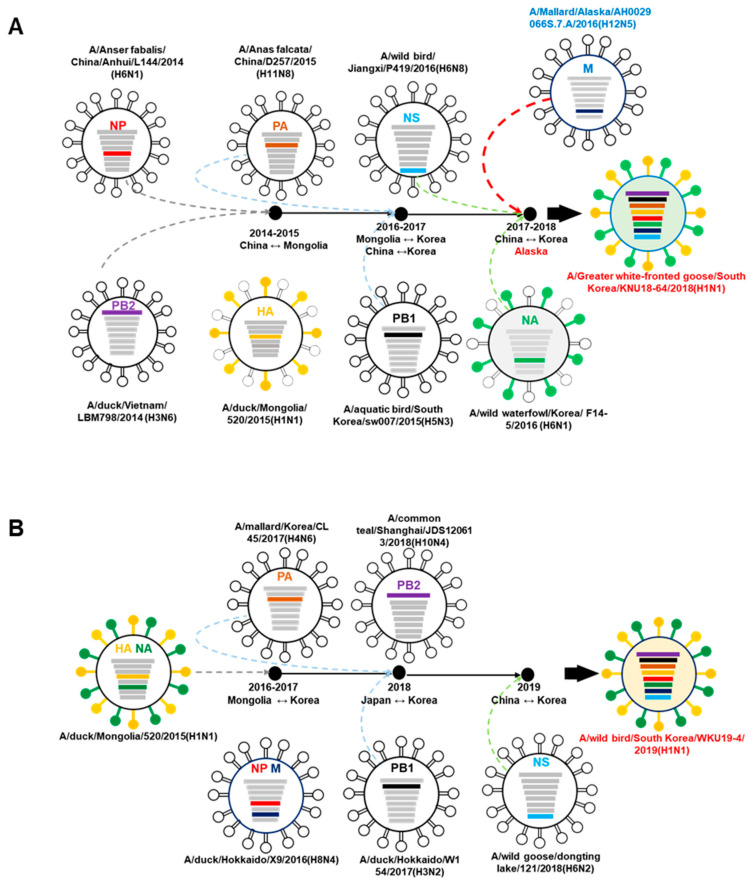
Hypothesis for the ancestor of each gene segment evolution of KNU18-64 (A/*Greater white-fronted goose*/South Korea/KNU18-64/2018(H1N1)) (**A**) and WKU19-4 (A/wild bird/South Korea/WKU19-4/2019(H1N1)) (**B**) strains.

After tracking each gene segment ancestor of two isolates KNU18-64 (H1N1) and WKU19-4 (H1N1) by a combination of the results obtained by the phylogenetic trees and genomic homology, we developed a hypothesis for the ancestor of each gene segment as shown in Figure 3. The ancestors of PB2 and NP gene of KNU18-64 isolate was identified in 2014 in China and Vietnam, they may have been introduced into China-Mongolia by migratory wild-bird during 2014–2015. In here, the reassorment may have happened with the donor virus A/duck/Mongolia/520/2015 (H1N1) (MN520-H1N1) before the movement of wild-bird to Korea during migration season 2016–2017. During this miratory season into Korea, MN520-H1N1 virus may have donated HA and NA gene to A/hooded crane/Korea/1176/2016(H1N1). The intercontinental migration of the wild bird from the East Asian–Australian Flyway (EAAF) and the West Pacific Flyway may have been involved in the transmission of the MP gene from Alaska strain into Korea in 2017–2018, and KNU18-64 may have been generated during this period.

Meanwhile, WKU19-4 may have been generated later with the reasorment of viruses from Japan-China with Korea strains during migration season 2018–2019.

### 3.3. Molecular Characterization of the H1N1 Isolate

The amino acids in the HA protein (positions 94, 116, 121, 134, 139, 142, 221, and 222; H5 numbering) represent adaptive mutations that enable the virus to bind preferentially to α-2,6-linked sialic acid and enhance viral fusion [33,34,35,36,37].

All the isolates investigated in the present study are characterized as low pathogenicity avian influenza viruses, harboring the amino acid sequence PSIQRS↓GLF. In addition to the HA cleavage site, the mutation sites for efficient binding to avian-like α-2,3-linked sialic acid receptors and the NA stalk region non-deletion sites supported the low-pathogenicity of these isolates (Table 2) [38]. However, the NA from both of our isolates contained isoleucine at positions 26 and 223, and asparagine at position 373, indicating increased virulence in mammals [39,40,41,42,43].

The analysis data of the internal protein segment mutation sites are presented in Table 3, which indicates that both KNU18-64 and WKU19-4 contain mammalian pathogenicity-related mutations which are present in the H1N1 isolates in both swine and human.

In addition, both isolates have three distinct mutation sites at PB1-F2 and PA, implicating that they might possess different virulence. For instance, asparagine at the amino acid position 66 in the PB1-F2 of KNU18-64 is substituted with serine in that position of WKU19-4, leading to increased virulence in mammal. Meanwhile, KNU18-64 isolate carried unique mutation of PA at position 328, implicating the higher virulence of WKU19-4 over KNU18-64.

However, multiple unique mutations present in human- and swine H1N1 (PB2-A588I/V, PB2-GQ590/591SR/K, PA-L336M, PA-F277S, PA-K356R, NP-R305K, NP-F313V, NP-I353V, NP-Q357K, M1-V15I, M1-A166V, M2-S31N, M2-L55F, NEP/NS2-M31I) were not found in both isolates, supporting their still low pathogenic traits.

### 3.4. Replication of H1N1 in Mammalian Cells

**Figure 4:**
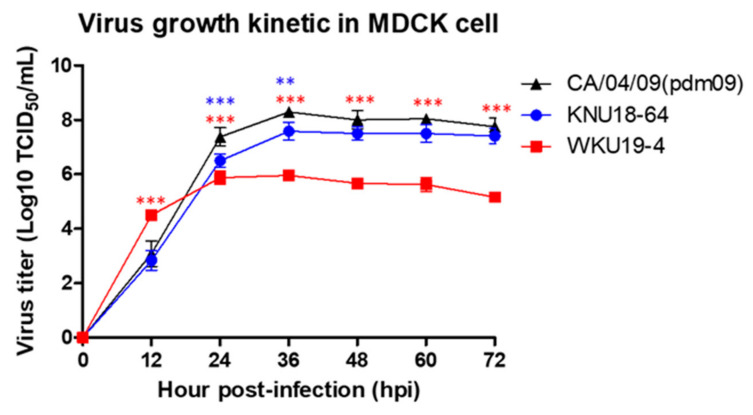
In vitro growth properties of KNU18-64 (A/*Greater white-fronted goose*/South Korea/KNU18-64/2018(H1N1)) and WKU19-4 (A/wild bird/South Korea/WKU19-4/2019(H1N1)) in Madin–Darby canine kidney (MDCK) cells. Virus titers were determined by the 50% tissue culture infectious dose (TCID_50_) assay. The cell monolayers were infected with the viruses at a multiplicity of infection (MOI) rate of 0.01, and incubated for 72 h. Every 12 h, the virus titers were determined by a TCID_50_ assay, and an enzyme-linked immunosorbent assay (ELISA) was performed with anti-influenza nucleoprotein to detect infected cells. The virus titers are the means ± standard deviations (SD) (n = 3), **, *p* < 0.01; ***, *p* < 0.001.

Because the genetic information implied that the two new reassortments of H1N1 would increase replication efficiency, the viral replication of these two isolates was tested in vitro. To evaluate the growth kinetics of the two isolates, a human-origin virus A/California/04/2009(H1N1), which infects other mammals was used as a control. The control H1N1 strain replicated more efficiently in MDCK cells than KNU18-64 (A/*Greater white-fronted goose*/South Korea/KNU18-64/2018(H1N1)) or WKU19-4 (A/wild bird/South Korea/WKU19-4/2019(H1N1)) (Figure 4). There is a dramatic increase in the viral titer after 12h post-inoculation of WKU19-4 to 4.5 log_10_ (TCID_50_/mL), whereas KNU18-64 has similar pattern with A/California/04/09. The raw data of the TCID50 assay are provided in Figure S2. This result may be explained by the molecular characteristic, the mutations in WKU19-4(H1N1) at amino acid position 66, in which asparagine is substituted with serine in the PB1-F2 protein, and a new substitution at position 321 of the PA segment, in which asparagine is substituted with isoleucine, indicate increasing virulence in mammals and increasing polymerase activity.

### 3.5. Pathogenicity in Mice

**Figure 5:**
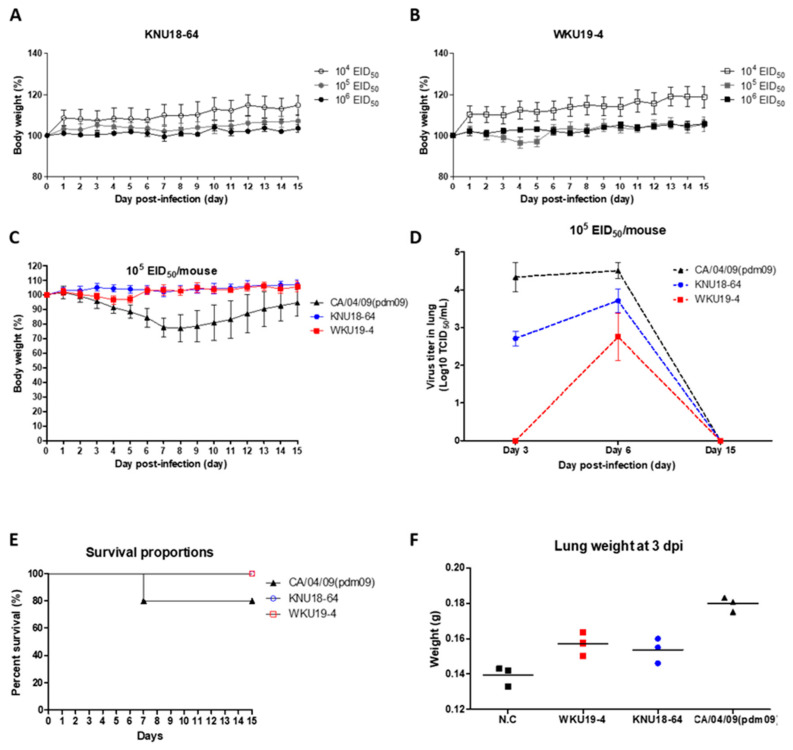
Pathogenicity of the two H1N1 isolates in vivo. For each isolate, BALB/c mice were intranasally infected with different EID_50_ concentrations of the virus (10^4^, 10^5^, and 10^6^ EID_50_/mouse). Changes to the weights of the mice are shown for KNU18-64 (**A**) and WKU18-4 (**B**), and the survival rates were noted. The BALB/c mice were intranasally challenged with 10^5^ EID_50_ of each virus, and a H1N1 (2009) strain isolated from humans (CA/04/09(pdm09)) was used as a control virus. Weights of the mice (**C**); virus titers in the lung (**D**); survival rates (**E**); mean of lung weights (**F**) (*n* = 3); Body weights are presented as percentages of the original weight (*n* = 5).

As previously described, the mouse model is widely used to determine the pathogenic potential of the influenza virus in mammals [89]. To assess the pathogenic potential of the new isolate in mammals, multiple concentrations of the virus (from 10^4^ to 10^6^ EID_50_/mouse) were used to determine the virulence of each new H1N1 isolate in the mice. After intranasal inoculation with these virus titers, changes in the body weights and survival rates of the 6-week-old female BALB/c mice were monitored for 15 dpi (Figure 5A,B). As shown in Figure 5A,B, the body weights of the mice did not decrease in either of the KNU18-64- or WKU19-4 infected groups. The infected mice displayed no severe clinical signs, such as ruffled fur, depression, or labored breathing, and each mouse was still alive at 15 dpi (data not shown).

To determine the virulence of the new H1N1 isolates in mammalian host, presence of each virus was detected in the lung of mice. Of all the mice infected with the control CA/04/09(pdm09) (H1N1), or challenged with KNU18-64 or WKU19-4, there was only significant weight loss in the control group CA/04/09 (Figure 5C), two mice died at 7 dpi in the control group (Figure 5E). In contrast, all the mice in the KNU18-64- and WKU19-4-infected groups had decreased slightly in weight by 3 dpi (e.g., the mice in the WKU19-4 group retained 98.97% of their body weight), but they quickly recovered and all the mice survived for at least 15 days.

Viral load shedding in the lungs of the mice at 3, 6, and 15 dpi was determined by TCID_50_ assays, and the results are presented in Figure 5D. As shown, the mice in the control group CA/04/09 had significantly higher virus titers in their lungs than the mice in the KNU18-64 or WKU19-4 groups at 3 dpi (4.33 ± 0.31; 2.71 ± 0.16; 0 log_10_ (TCID_50_/mL), respectively) and at 6 dpi (4.5 ± 0.17; 3.71 ± 0.25; 2.75 ± 0.51 log_10_(TCID_50_/mL), respectively), and no viruses were detected at 15 dpi in any of the groups. The raw data of the TCID_50_ assay are provided in Figure S3.

**Figure 6:**
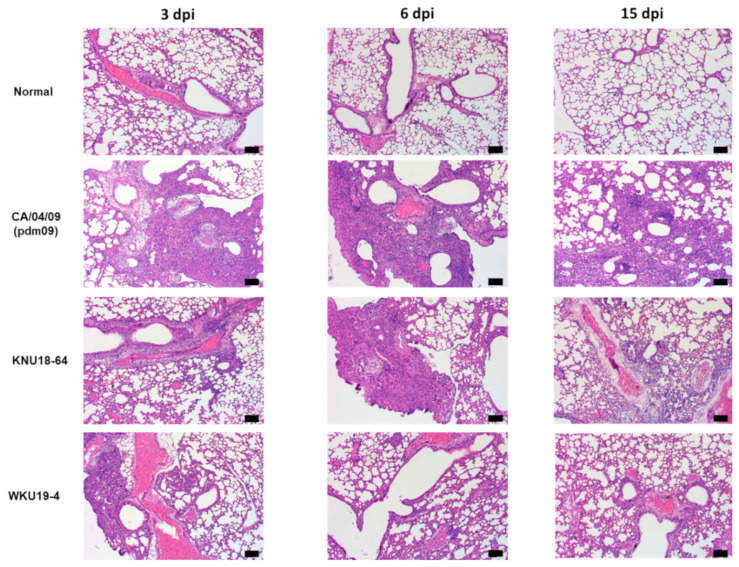
Histology of lung inflammation determined by hematoxylin and eosin (H&E) staining. For each isolate, BALB/c mice were intranasal infected with EID_50_ concentrations of the virus at 10^5^ EID_50_/mouse. The uninfected control (normal); CA/04/09(pdm09) (H1N1)-, KNU18-64 (H1N1)-, and WKU19-4 (H1N1)-infected mouse lungs were collected and stained with hematoxylin and eosin (H&E) at 3, 6, and 15 days post-infection (dpi) (scale bar, 100 µm; original magnification × 100).

It is worth noting that there were no significant changes in body weight, despite the mutation characteristics of the new isolates. Therefore, we carried out histopathological examinations of the lungs of the mice in each group. The H&E-stained sections of the KNU18-64-, WKU19-4-, and CA/04/09(pdm09) H1N1-infected lungs revealed that neutrophils had penetrated the alveolar air spaces at 3 and 6 dpi (Figure 6). The KNU18-64- and WKU19-4-infected lungs exhibited cellular accumulation compared to the lungs of the normal mice at 6 dpi. Furthermore, the lungs of the KNU18-64- and WKU19-4-infected mice increased significantly in weight compared to those of the normal mice at 3 dpi (Figure 5F) although it becomes non-significant at 6 and 15 dpi as presented data in Figure S4. The real lung morphology of this experiment is presented in the Figure S5.

## 4. Discussion

As a precaution, the prevalence of a newly emergent strain should be reported early, and broad surveillance of the H1N1 strain is a requirement. In the present study, we analyzed the molecular characteristics and virulence of two new H1N1 influenza virus strains of avian origin, which were isolated from the feces of migrating wild birds in Korea during the winter of 2018–2019.

Recently, an avian-origin H1N1 strain (A/SK14/2014) was reported in Korea. Compared to this isolate, the HA gene from both of our current isolates (KNU18-64 and WKU19-4) are closely related to a Mongolian strain (H1N1-2015) of avian origin (Figure 2A). Meanwhile, the HA gene from the previously reported strain is closer to a Chinese H1N4 virus of avian origin [44].

Phylogenetic data analysis revealed that the NA gene segment of the WKU19-4 isolate closely resembles that of a Bangladesh strain from 2017, whereas KNU18-64 closely resembles the Korea avian influenza virus H6N1 from 2017 [90].

According to the phylogenetic tree of the NA gene, the American avian influenza virus H6N1 strains from 2015 appears to be closest to that of KNU18-64 strain. Interestingly, MP gene of KNU18-64 is closest to those in the Alaskan–American strains, indicating that the KNU18-64 isolate is related to the Alaskan–American influenza A viruses of avian origin, as previously reported [28,91].

Analysis of the TCID50, plaque assay, and/or results of quantitative reverse-transcription PCR with mathematical modeling is a powerful approach to the detailed characterization of viral replication in vitro [22,28]. In the present study, the new isolates exhibited different degrees of virulence, which can be explained by their molecular mutation characteristics, as demonstrated by the kinetic growth dynamics of each virus shown. The WKU19-64 virus appeared to replicate rapidly in the mammalian MDCK cells, and its replication decreased dramatically and stabilized after 1 dpi. In contrast, the KNU18-64 isolate exhibited dynamic kinetic growth similar to that of the positive control infected with the human-origin H1N1 (the CA/04/09 group). This may have been because the WKU19-4 isolate also contains a new substitution of asparagine for isoleucine at position 321 of the PA segment, which may be related to the increase in polymerase activity that caused the virus to replicate dramatically at 12 hpi.

As shown in Figure 6, both of the new isolates exhibited moderate pathogenicity in mice, with significantly lower virus titers in the lung after 3 and 6 dpi compared to the control, which was infected with human-origin H1N1 (the CA/04/09(pdm09) group). In the case of WKU19-4, the virus was only observed at 6 dpi, with the lowest titer of 2.75 ± 0.51 log_10_ (TCID_50_/mL). The KNU18-64 isolate appeared more pathogenic in mice; the kinetic virus titers were significantly higher than in WKU19-4 at both 3 and 6 dpi (2.71 ± 0.16 log_10_ (TCID_50_/mL) and 3.71 ± 0.25 log_10_ (TCID_50_/mL)], respectively. This result appears to corroborate the results of the molecular characterization section; the KNU18-64 isolate carries two individual mutations in the PA protein (phenylalanine is substituted with serine at position 227, and lysine is substituted with arginine at position 328), which lead to increased virulence in and the adaptation to mammals.

The present study suggests that the two H1N1 AIV isolates found in Korean both in the same season generated by reassortment events occurred following the co-circulation of Eurasian as well as Alaskan strains. Therefore, intensive surveillance of low pathogenic AIVs in wild migratory birds is an important tool to detect major AIVs which could threaten human health in Korea.

## Figures and Tables

**Table 1:** Virus strains from the GenBank database with the highest nucleotide identities with the two H1N1 isolates investigated in the present study.

	A/*Greater White-Fronted Goose*/South Korea/KNU18-64/2018(H1N1)				A/Wildbird/South Korea/WKU19-4/2019(H1N1)			
Gene Segment	Genebank ID	Reference Strain Accession ID	Highest Similarly Strain Reference	Per. Ident (%)	Genebank ID	Reference Strain Accession ID	Highest Similarly Strain Reference	Per. Ident (%)
PB2	MN584878.1	LC053481.1	A/duck/Vietnam/LBM798/2014(H3N6)	99.08(2280/2280)	MT821115.1	MN049531.1	A/common teal/Shanghai/JDS120613/2018(H10N4)	99.259(2259/2280)
		MN171439.1	A/duck/Jiangsu/S1665/2015(H5N3)	98.86(2293/2280)		MK592490.1	A/duck/Hokkaido/56/2017(H12N2)	98.86(2280/2280)
		KU881717.1	A/Anseriformes/Anhui/L259/2014(H1N1)	98.86(2280/2280)		MK592458.1	A/duck/Akita/51019/2017(H5N3)	98.64(2280/2280)
PB1, PB1F2	MN584879.1	MG386194.1	A/aquatic bird/South Korea/sw007/2015(H5N3)	99.08(2274/2274)	MT821116.1	MK592547.1	A/duck/Hokkaido/W154/2017(H3N2)	99.56(2274/2274)
		MH130136.1	A/wild waterfowl/Korea/F56-1/2017(H6N2)	98.77(2274/2274)		MK592531.1	A/duck/Hokkaido/W144/2017(H3N2)	99.56(2274/2274)
		KX121186.1	A/bean goose/Hubei/SZY200/2016(H11N9)	98.77(2274/2274)		MK592515.1	A/duck/Hokkaido/W105/2017(H5N2)	99.56(2274/2274)
PA	MN584880.1	MH547049.1	A/Anas falcata/China/D257/2015(H11N8)	98.93(2151/2151)	MT821117.1	MH579404.1	A/mallard/Korea/CL45/2017(H4N6)	99.12(2151/2151)
		MK943289.1	A/duck/Viet Nam/HN-2525/2015(mixed)	98.47(2151/2151)		MH727479.1	A/wild goose/dongting lake/121/2018(H6N2)	98.98(2151/2151)
		KF454809.1	A/mallard/Mongolia/1551/2010(H3N1)	98.47(2151/2151)		MN703036.1	A/duck/Cambodia/10T-24-1-D14/2018(mixed)	98.31(2151/2151)
HA	MN584881.1	LC121396.1	A/duck/Mongolia/520/2015(H1N1)	98.53(1726/1701)	MT821118.1	LC121396.1	A/duck/Mongolia/520/2015(H1N1)	99.06(1701/1701)
		KY402065.1	A/hooded crane/Korea/1176/2016(H1N1)	98.12(1701/1701)		LC121276.1	A/duck/Mongolia/154/2015(H1N2)	98.88(1701/1701)
		LC121276.1	A/duck/Mongolia/154/2015(H1N2)	98.12(1732/1701)		KY402065.1	A/hooded crane/Korea/1176/2016(H1N1)	98.65(1701/1701)
NP	MN584882.1	KT717283.1	A/Anser fabalis/China/Anhui/L144/2014(H6N1)	99.53(1497/1497)	MT821119.1	MK978905.1	A/duck/Hokkaido/X9/2016(H8N4)	99.2(1497/1497)
		KR010412.1	A/goose/Inner Mongolia/IM-192/2014(H5N1)	99.4(1497/1497)		KF259820.1	A/common teal/Hong Kong/MPM1670/2011(H7N7)	99.13(1497/1497)
		KT717243.1	A/Anser fabalis/China/Anhui/S104/2014(H6N2)	99.33(1497/1497)		KF259825.1	A/wild waterfowl/Hong Kong/MPL1006/2011(H7N7)	99.06(1497/1497)
NA	MN584883.1	MH130116.1	A/wild waterfowl/Korea/F14-5/2016(H6N1)	98.94(1429/1410)	MT821120.1	LC121398.1	A/duck/Mongolia/520/2015(H1N1)	98.94(1410/1410)
		MH130132.1	A/bean goose/Korea/F54-8/2017(H6N1)	98.87(1424/1410)		MH791653.1	A/duck/Bangladesh/34191/2017(H3N1)	98.58(1410/1410)
		MH130124.1	A/bean goose/Korea/F27-6/2017(H6N1)	98.87(1410/1410)		KY402067.1	A/hooded crane/Korea/1176/2016(H1N1)	98.51(1410/1410)
M2, M1	MN584884.1	MN253720.1	A/Mallard/Alaska/AH0029066S.7.A/2016(H12N5)	98.68(1007/982)	MT821121.1	MK978907.1	A/duck/Hokkaido/X9/2016(H8N4)	99.49(982/982)
		KY130619.1	A/green-winged teal/Alaska/292/2011(H3N8)	98.07(995/982)		KY635666.1	A/duck/Bangladesh/26980/2015(H7N9)	99.19(982/982)
		CY120628.1	A/American wigeon/California/3180/2010(H5N2)	98.07(995/982)		KY635509.1	A/duck/Bangladesh/27042/2015(H7N9)	99.18(982/982)
NEP, NS1	MN584885.1	KX867861.1	A/wild bird/Jiangxi/P419/2016(H6N8)	99.16(864/838)	MT821122.1	MN483241.1	A/White-fronted Goose/South Korea/KNU18-119/2018(H7N7)	99.64(855/838)
		KY635798.1	A/black-tailed godwit/Bangladesh/24734/2015(H7N5)	99.05(849/838)		MH727484.1	A/wild goose/dongting lake/121/2018(H6N2)	99.64(838/838)
		KT266946.1	A/duck/Guangxi/113/2012(H6N8)	99.28(864/838)		MT090541.1	A/duck/Bangladesh/38292/2019(H2N2)	99.52(852/838)

**Table 2:** Comparison between the hemagglutinin (HA) receptor-binding sites and neuraminidase (NA) of the two novel avian H1N1 isolates and those of human, swine, and avian H1N1 isolates.

Virus Strain		HA Receptor-Binding Residues (H5 Numbering)								NA					
	Cleavage Sites	D/E94N	I116M	S121N	A134V	G139R	S142G	D221G/N	Q222L	Deleted Range from 50–70	M26I	I106V	T223I	K/S373A/N	G394D
A/wild bird/South Korea/WKU2019-4/2019(H1N1)	PSIQRS↓GLF	E	I	S	A	G	S	G	Q	No deletion	I	I	I	N	G
A/*Greater white-fronted goose*/South Korea/KNU18-64/2018	PSIQRS↓GLF	E	I	S	A	G	S	G	Q	No deletion	I	I	I	N	G
A/wild bird/Korea/SK14/2014 ^a^	PSIQRS↓GLF	E	I	S	A	G	S	G	Q	No deletion	I	I	I	N	G
A/duck/Mongolia/520/2015 ^b^	PSIQRS↓GLF	E	I	S	A	G	S	G	Q	No deletion	I	I	I	N	G
A/California/04/2009 ^c^	PSIQRS↓GLF	D	I	S	A	G	S	D	Q	No deletion	I	V	I	N	G
A/swine/Shandong/1207/2016 ^d^	PSIQRS↓GLF	E	I	S	A	G	S	E	Q	No deletion	I	V	I	N	G

^a^ The representative avian H1N1 strain that was isolated from the feces of migratory birds in the Republic of Korea during the winter of 2014–2015 [44]. ^b^ Mongolia strain with HA and NA sequences that most closely resemble the corresponding sequences in the two isolates of the present study. ^c^ Human pandemic H1N1 isolate. ^d^ H1N1 strain that originated in swine [45].

**Table 3:** Molecular characteristic of two novel isolate influenza A (H1H1) virus.

Viral Protein	Amino Acid	W4	K64	SK14	M520	CA/04/09	SW/1207	Comments	Reference
PB2	E627K	E	E	E	E	E	E	Mammalian host adaptation, Human host marker, Enhanced polymerase activity, Increased virulence in mammals	[46,47,48,49,50,51]
	T63I (with PB1 M677T)	I	I	I	I	I	I	Enhanced polymerase activity, Increased virulence in mice	[51]
	L89V	V	V	V	V	V	V	Enhanced polymerase activity, Increased virulence in mice	[52]
	K251R	R	R	R	R	R	R	Increased virulence in mice	[41]
	T271A	T	T	T	T	A	A	Human host marker (host-specific polymerase activity)	[53]
	G309D	D	D	D	D	D	D	Enhanced polymerase activity, Increased virulence in mice	[52]
	T339K	K	K	K	K	K	K	Enhanced polymerase activity, Increased virulence in mice	[52]
	R/Q355K	R	R	R	R	R	K	Increased virulence in mammals	[42]
	Q368R	R	R	R	R	R	R	Increased polymerase activity, Increased virulence in mammals	[54]
	H447Q	Q	Q	Q	Q	Q	Q	Increased polymerase activity, Increased virulence in mammals	[54]
	R477G	G	G	G	G	G	G	Enhanced polymerase activity, Increased virulence in mice	[52]
	I495V	V	V	V	V	V	V	Enhanced polymerase activity, Increased virulence in mice	[52]
	A588I/V	A	A	A	A	**T**	**I**	Human host marker (host-specific polymerase activity)	[53]
	GQ590/591SR/K	GQ	GQ	GQ	GQ	**SR**	**SR**	Increased polymerase activity, Human host adaptation	[55]
	Q591K	Q	Q	Q	Q	R	R	Increased virulence in mammals	[56]
	A/S674T	A	A	A	A	A	A	Human host marker	[22]
	A676T	T	T	T	T	T	T	Enhanced polymerase active, Increased virulence in mice	[52]
	D701N	D	D	D	D	D	D	Increased polymerase activity, Increased virulence in mammals, Mammalian host marker	[55,57,58]
	K702R	K	K	K	K	K	K	Human host marker	[48,49,57]
PB1	D3V	V	V	V	V	V	V	Increased polymerase activity, Increased virulence in mammals	[59]
	L13P	P	P	P	P	P	P	Increased polymerase activity, Increased virulence in mammals, Mammalian host marker	[58]
	R207K	K	K	K	K	K	K	Increased polymerase activity in mammalian cells	[60]
	K328N	N	N	N	N	N	N	Increased polymerase activity, Increased virulence in mammals	[61]
	S375N	N	N	N	N	N	N	Increased polymerase activity, Increased virulence in mammals, Human host marker	[49]
	H436Y	Y	Y	Y	Y	Y	Y	Increased polymerase activity and virulence in mallards, ferrets and mice	[60]
	A469T	T	T	T	T	T	T	Conferred in contact transmissibility in guinea pigs.	[62]
	L473V	V	V	V	V	V	V	Increased polymerase activity and replication efficiency	[63]
	V652A	A	A	A	A	A	A	Increased virulence in mice	[41]
	M677T	T	T	T	T	T	T	Pathogenic in mice	[51]
PB1-F2	N66S	S	N	N	S	-	-	Increased virulence in mammals	[64]
PA	S37A	A	A	A	A	A	A	Significantly increased viral growth and polymerase activity in mammalian cells	[65]
	S224P (with N383D)	S	S	S	S	**P**	S	Enhanced the pathogenicity and viral replication of H5N1 virus in mice	[66]
	H266R	R	R	R	R	R	R	Increased polymerase activity. Increased virulence in mammals and birds	[67,68]
	F277S	F	S	S	S	**H**	**H**	Contributed to the virulence and mammalian adaptation	[67]
	C278Q	Q	Q	Q	Q	Q	Q	Adapt to mammalian hosts	[69]
	N321K	I *	N	N	N	N	**V**	Increased polymerase activity	[37]
	K328R	K	R	K	K	K	K	Increased virulence in mice	[40]
	L336M	L	L	L	L	**M**	**M**	Increased virulence in mammals	[70]
	K356R	K	K	K	K	**R**	**R**	Increased virulence in mammals and mice	[71]
	N383D (with S224P)	D	D	D	D	D	D	Enhanced the pathogenicity and viral replication of H5N1 virus in mice	[66]
	S409N	S	S	S	S	N	N	Enhanced Transmission, Human host marker	[70]
	S/A515T	T	T	T	T	T	T	Increased polymerase activity. Increased virulence in mammals and birds	[60]
PA-X	R195K	R	R	R	R	-	K	Increased virulence in mammals	[72]
HA	Cleavage site	PSIQRS↓GLF	PSIQRS↓GLF	PSIQRS↓GLF	PSIQRS↓GLF	PSIQRS↓GLF	PSIQRS↓GLF		[39]
	D/E94N	E	E	E	E	**D**	E	Increased virus binding to 2,6, Enhanced virus fusion	[33]
	I116M	I	I	I	I	I	I	Potential to alter the virulence of H1N1pdm09 in swine	[34]
	S121N	S	S	S	S	S	S	Increased virus binding to 2,6, Increased replication in mammals	[35]
	A134V	A	A	A	A	A	A	Increased virus binding to 2,6	[36]
	G139R	G	G	G	G	G	G	Increased virus binding to 2,6	[37]
	S142G	S	S	S	S	S	S	Potential to alter the virulence of H1N1pdm09 in swine	[34]
	D221G/N	G	G	G	G	**D**	**E**	Change in receptor binding	[35]
	Q222L	Q	Q	Q	Q	Q	Q	Increased virus binding to 2,6	[73]
NA	M26I	I	I	I	I	I	I	Increased virulence in mice	[40]
	DELETED Range from aa 50–70	NO DELETION	NO DELETION	NO DELETION	NO DELETION	NO DELETION	NO DELETION	the adaptation of influenza viruses from wild aquatic birds to domestic chickens	[39]
	I106V	I	I	I	I	V	V	Increased virulence in mice	[41]
	R143K	K	K	K	K	K	K	Increased virulence in mammals and mice	[74]
	T223I	I	I	I	I	I	I	Increased virulence in mammals	[42,75,76]
	V241I	V	V	V	V	V	V	Contributed to the significantly higher baseline IC50 value obtained to oseltamivir for 2010/2011 viruses	[43]
	N248D	N	N	N	N	N	N	Increased virulence in mice	[41]
	H275Y	H	H	H	H	H	H	Increased virulence in mammals	[77]
	R293K	R	R	R	R	R	R	Highly resistant to the oseltamivir	[78]
	S354N	N	N	N	N	N	N	Increased virulence in mice	[79]
	K/S373A/N	N	N	N	N	N	N	Increased virulence in mammals	[80]
NP	V41I	I	I	I	I	I	I	Might contribute to viral transmissibility	[81]
	V105M	M	M	M	M	M	M	Contribute to the increased virulence of the H9N2	[82]
	D210E	E	E	E	E	E	E	Might contribute to viral transmissibility	[81]
	F253I	I	I	I	I	I	I	Results in attenuated pathogenicity of the virus in mice	[83]
	R305K	R	R	R	R	**K**	**K**	Human host marker	[48]
	F313V	F	F	F	F	**V**	**V**	Human host marker	[53]
	I353V	I	I	I	I	**V**	**V**	Increased virulence in mice	[41]
	Q357L/K (WITH PB2 I27K)	Q	Q	Q	Q	**K**	**K**	Increased virulence in mammals, Human host marker	[48]
M1	V15I/T	V	V	V	V	**I**	**I**	Increased virulence in mammals	[42]
	N30D	D	D	D	D	**S**	**S**	Increased virulence in mammals	[84]
	A166V	V	V	V	V	**A**	**A**	Contribute to the increased virulence of the H9N2.	[82]
M2	S31N L55F	S L	S L	S L	S L	**N** **F**	**N** **F**	resistance to the adamantane antivirals and are sensitive to oseltamivir and zanamivir Enhanced Transmission	[53] [85]
NS1	A/P42S	S	S	S	S	S	S	Increased virulence in mammals, Antagonism of IFNinduction	[86]
	D74N	D	D	D	D	**S**	**S**	Increased virulence in mammals	[87]
	T/D/V/R/A127N	N	N	N	N	N	N	Increased virulence in mammals	[86]
	V149A	A	A	A	A	A	A	Pathogenicity in mice, Antagonism of IFN induction	[88]
	N/G205R/K (with NEP/NS2 M51I, T48N; PB2 A469T)	S	S	S	S	**N**	**K**	Decreased IFN antagonism, Conferred enhanced in-contact transmissibility in guinea pigs	[62]
NEP/ NS2	M31I	M	M	M	M	M	**I**	Increased virulence in mammals	[86]

H3 numbering. W4: A/wild bird/South Korea/WKU2019-4/2019(H1N1); K64: A/*Greater white-fronted goose*/South Korea/KNU2018-64/2018; SK14: A/wild bird/Korea/SK14/2014; M520: A/duck/Mongolia/520/2015; CA/04/09: A/California/04/2009 infected-human isolate; SW/1207: A/swine/Shandong/1207/2016 Swine infected isolate; (-) Isolate that do not have PB1-F2, PA-X; (*) new mutation site; Sequence in Bold: mutation that only presented in the mammalian isolates; sequence in Underline: mutation that differs between isolates K64 and W4.

## Data Availability

The data presented in this study are available in this article and Supplementary Material.

## Supplementary Materials

The following are available online at https://www.mdpi.com/1999-4915/13/1/30/s1, Figure S1: The geographical location of two novel isolates A/Greater white-fronted goose/South Korea/KNU18-64/2018(H1N1) and A/wild bird/South Korea/WKU19-4/2019(H1N1) in Korea. Figure S2: Raw ELISA data of TCID50 assay for detect virus growth kinetic in MDCK cell. Figure S3. Raw ELISA data of TCID50 assay for viral load shedding in lung after 3 (A), 6 (B) and 15 (C) day post-infection. Figure S4. Mean lung weight at day 6 (A) and day 15 (B) post infection. Figure S5. Lungs from infected mouse at day 3, day 6, day 15 post infection.
