# Supplementary material for: Emergence of Novel Reassortant H1N1 Avian Influenza Viruses in Korean Wild Ducks in 2018 and 2019
Source: Viruses. 2020 Dec 26;13(1):30. doi: 10.3390/v13010030 (PMC7823676; doi:10.3390/v13010030)
Supplement: Supplementary file 1 [file viruses-13-00030-s001.pdf]

Supplementary data

Emergence of novel reassortant H1N1 avian influenza viruses in Korea wild duck in 2018 and 2019

Thuy-Tien Thi Trinh[1], Bao Tuan Duong[1], Anh Thi Viet Nguyen[1], Hien Thi Tuong[1], Vui Thi Hoang[1], Duong Duc Than[1], SunJeong Nam[2], Haan Woo Sung[3], Ki-Jung Yun[4], Seon-Ju Yeo[5, §], and Hyun Park[1, §]

Figure S1. The geographical location of two novel isolates A/Greater white-fronted goose/South Korea/KNU18-64/2018(H1N1) and A/wild bird/South Korea/WKU19-4/2019(H1N1) in Korea.

Figure S2. Raw ELISA data of TCID50 assay for detect virus growth kinetic in MDCK cell

Figure S3. Raw ELISA data of TCID50 assay for viral load shedding in lung after 3 (A), 6 (B) and 15 (C) day post-infection

Figure S4. Mean lung weight at day 6 (A) and day 15 (B) post infection

Figure S5. Lungs from infected mouse at day 3, day 6, day 15 post infection.

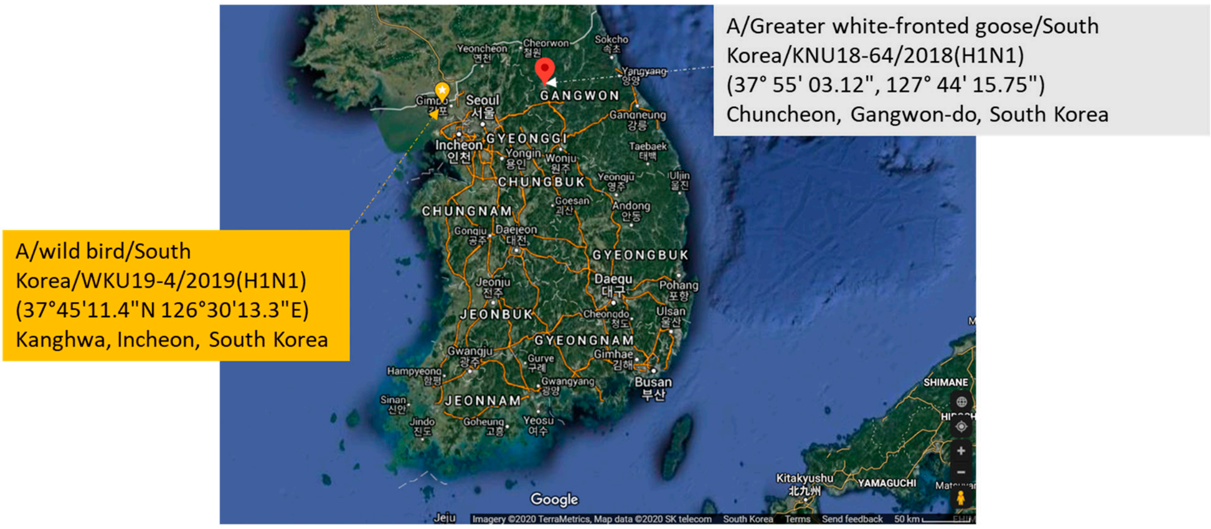

Fig. S1. The geographical location of two novel isolates A/Greater white-fronted goose/South Korea/KNU18-64/2018(H1N1) and A/wild bird/South Korea/WKU19-4/2019(H1N1) in Korea.

A

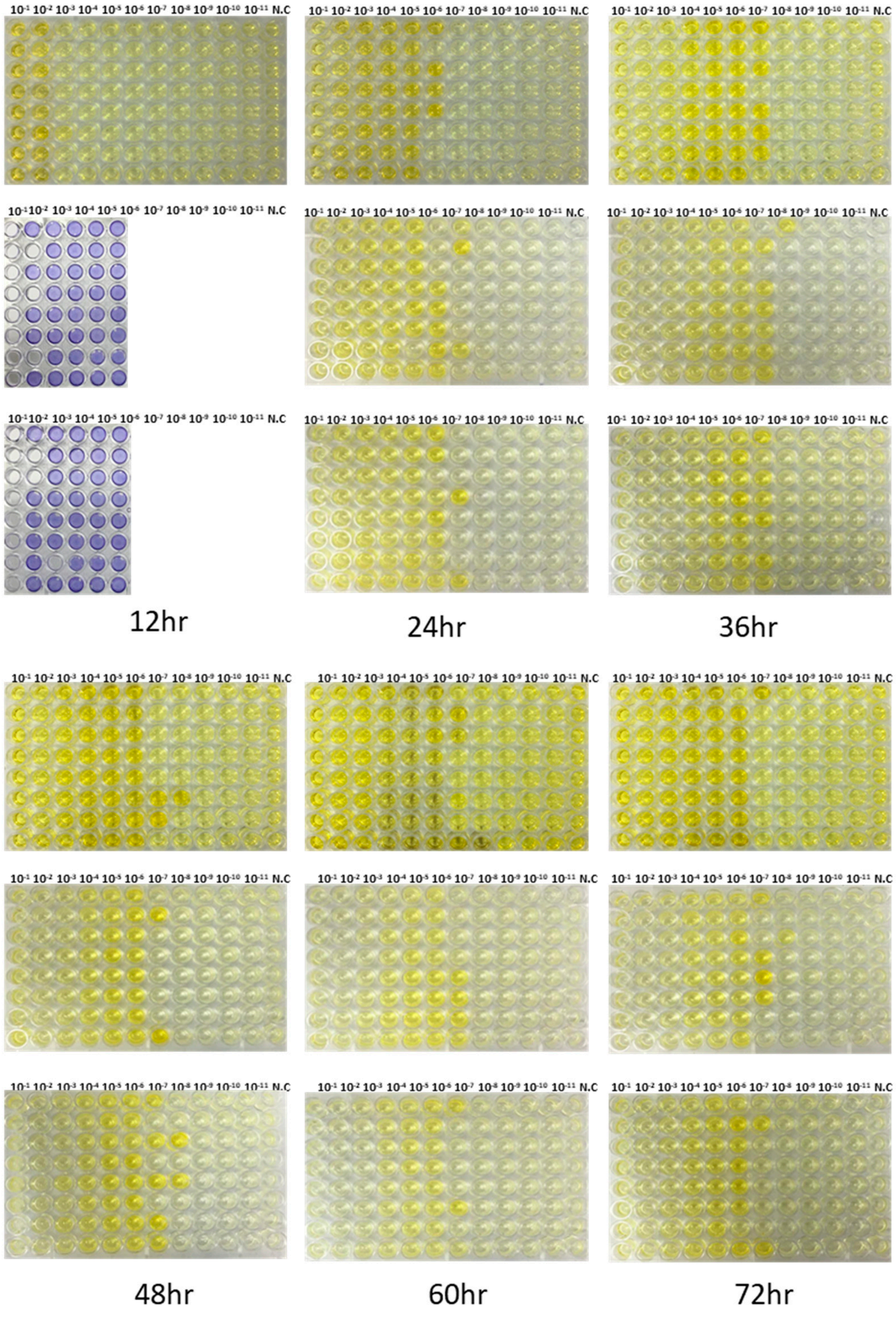
10-1 10-2 10-3 10-4 10-5 10-6 10-7 10-8 10-9 10-10 10-11 N.C
12hr
24hr
36hr
48hr
60hr
72hr

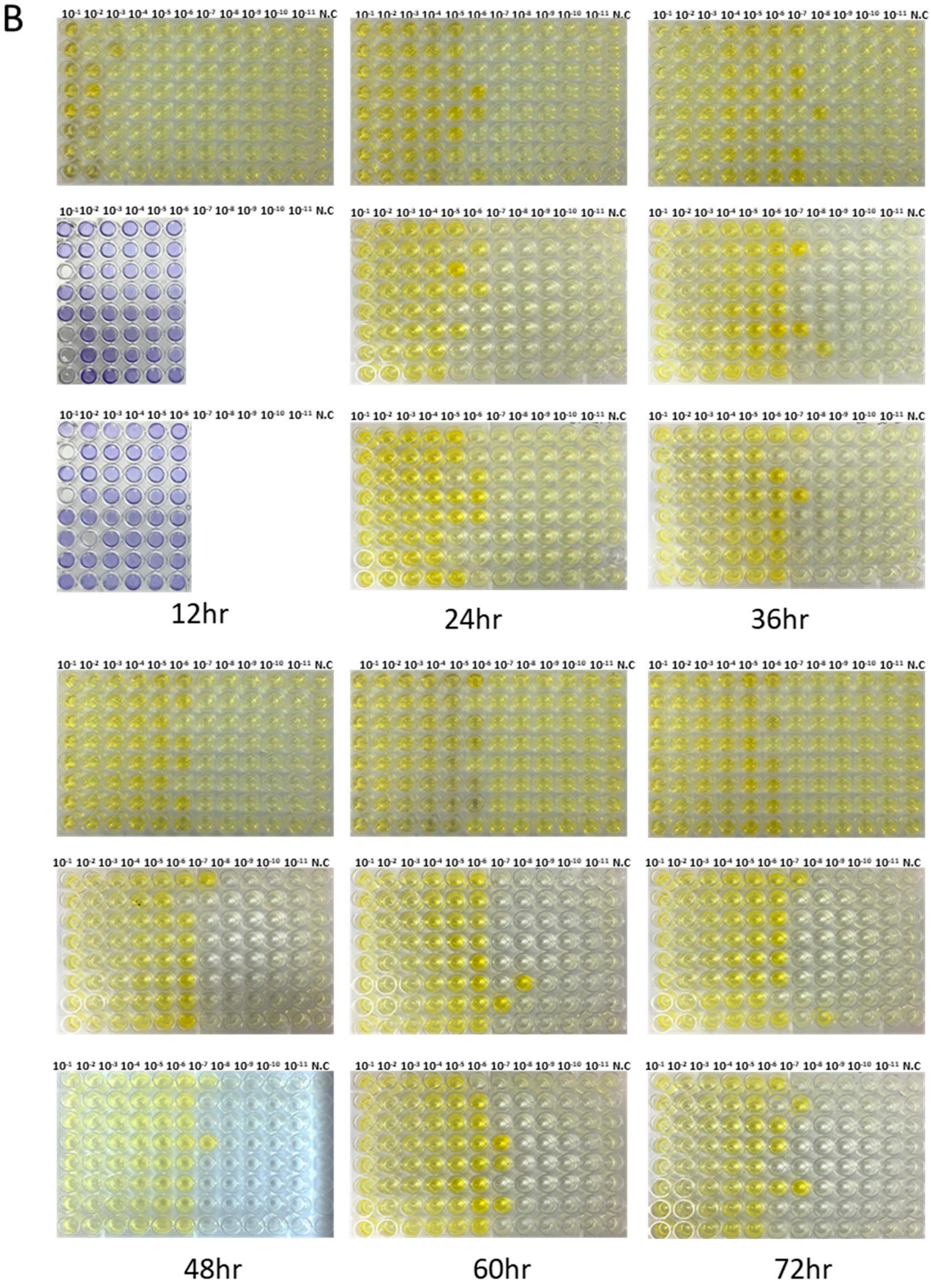
B
10^{-1} 10^{-2} 10^{-3} 10^{-4} 10^{-5} 10^{-6} 10^{-7} 10^{-8} 10^{-9} 10^{-10} 10^{-11} N.C
12hr
24hr
36hr
48hr
60hr
72hr

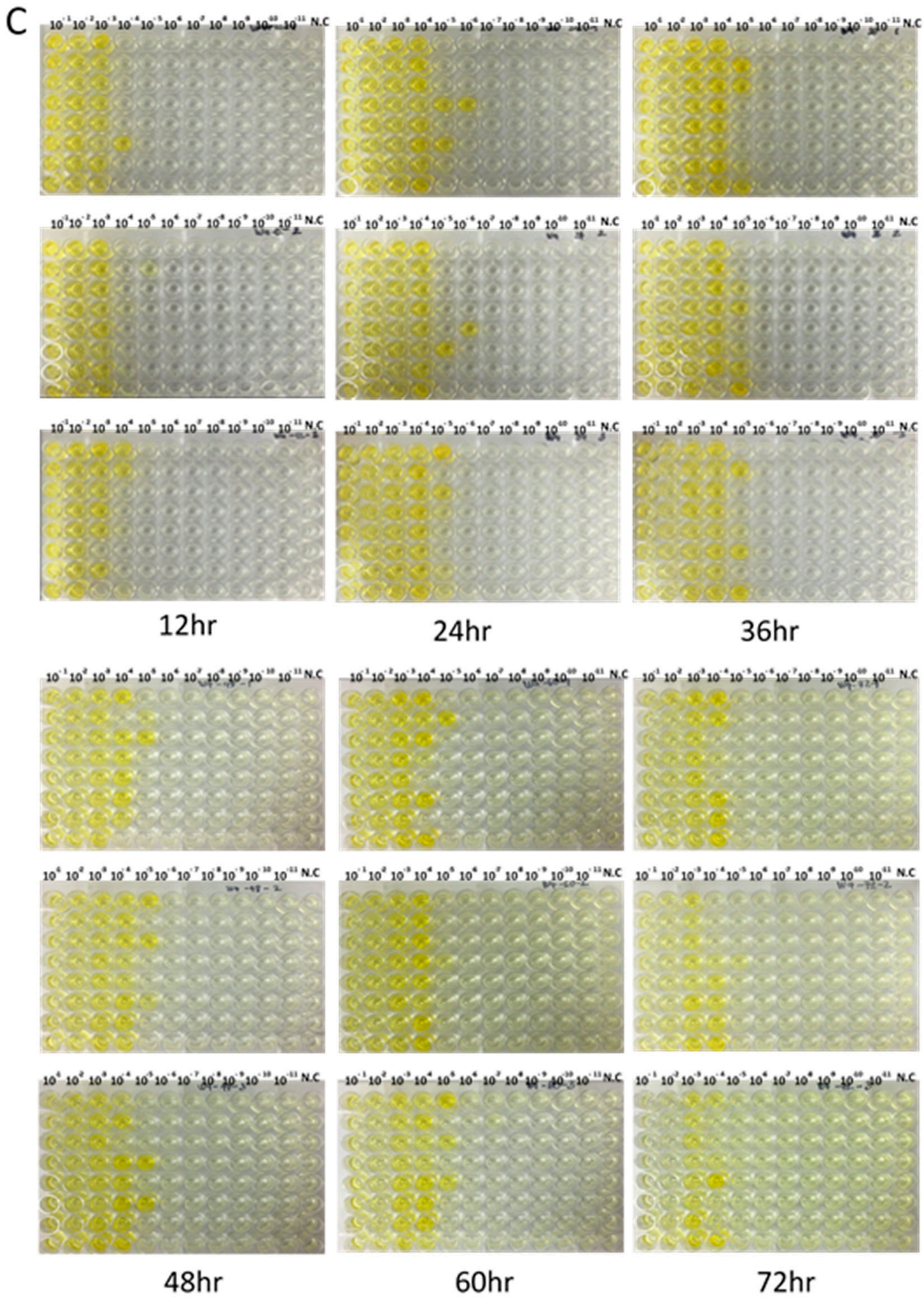

Figure S2. Raw ELISA data of TCID50 assay for detect virus CA/04/09 (pdm09) (A), KNU2018-64 (B), WKU2019-4 (C) growth kinetic in MDCK cell.

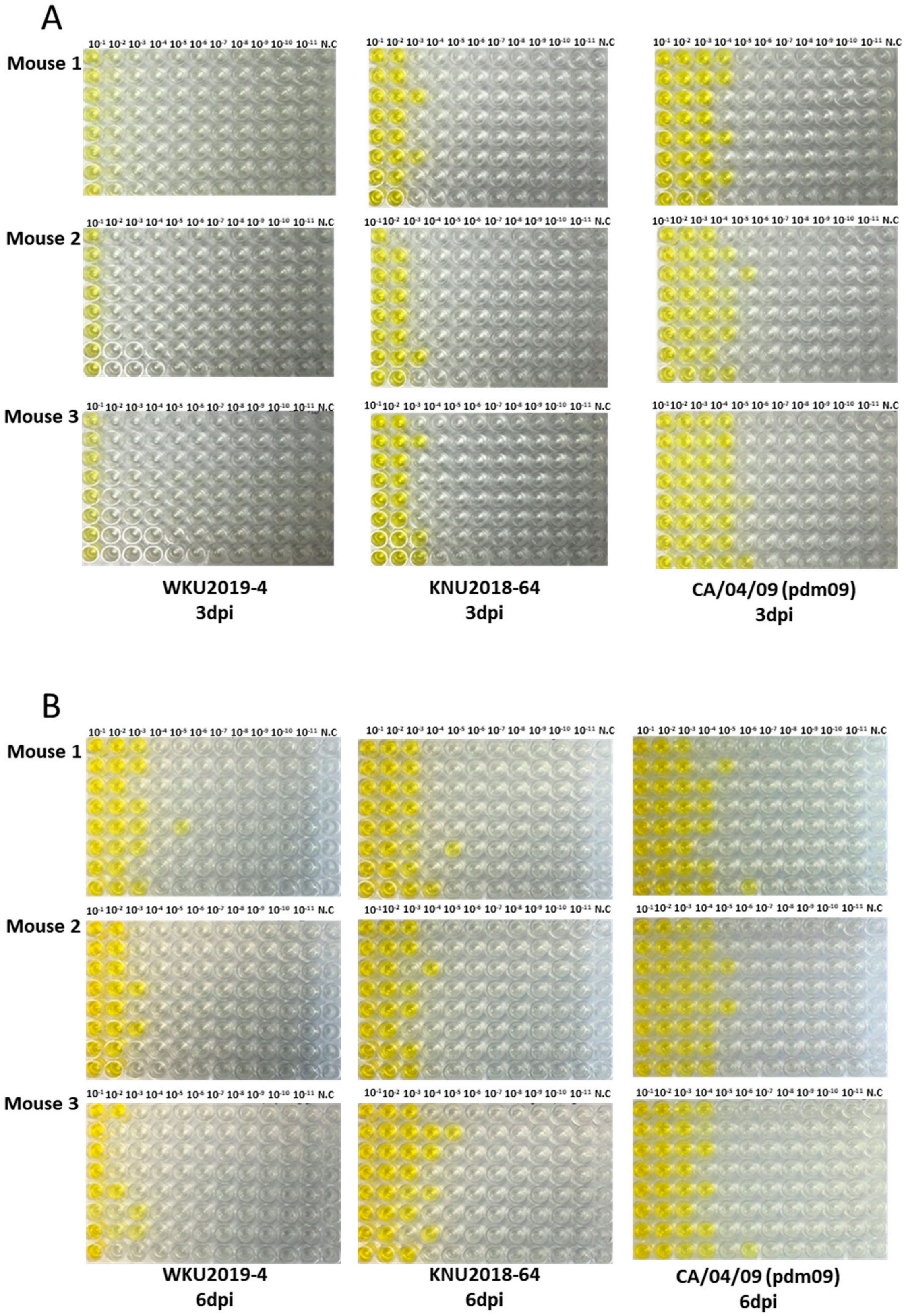
A
10^{-1} 10^{-2} 10^{-3} 10^{-4} 10^{-5} 10^{-6} 10^{-7} 10^{-8} 10^{-9} 10^{-10} 10^{-11} N.C
Mouse 1
Mouse 2
Mouse 3
WKU2019-4
3dpi
KNU2018-64
3dpi
CA/04/09 (pdm09)
3dpi
B
Mouse 1
Mouse 2
Mouse 3
WKU2019-4
6dpi
KNU2018-64
6dpi
CA/04/09 (pdm09)
6dpi

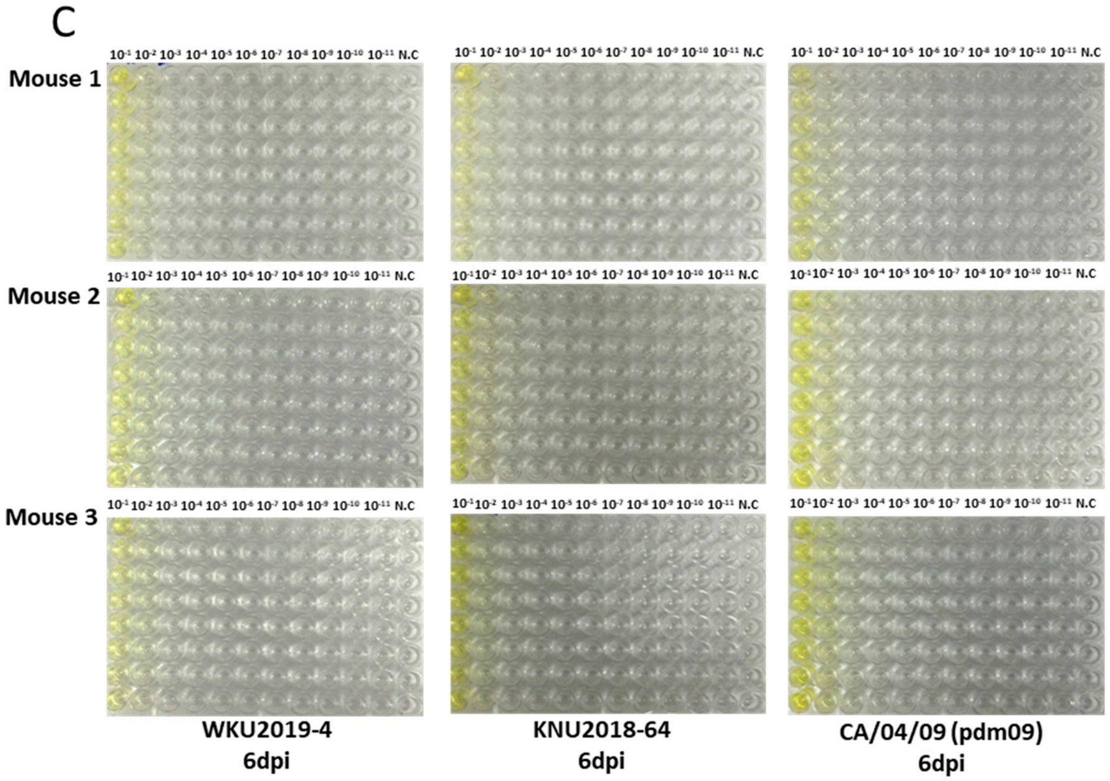

Figure S3. Raw ELISA data of TCID50 assay for viral load shedding in lung after 3 (A), 6 (B) and 15 (C) day post-infection

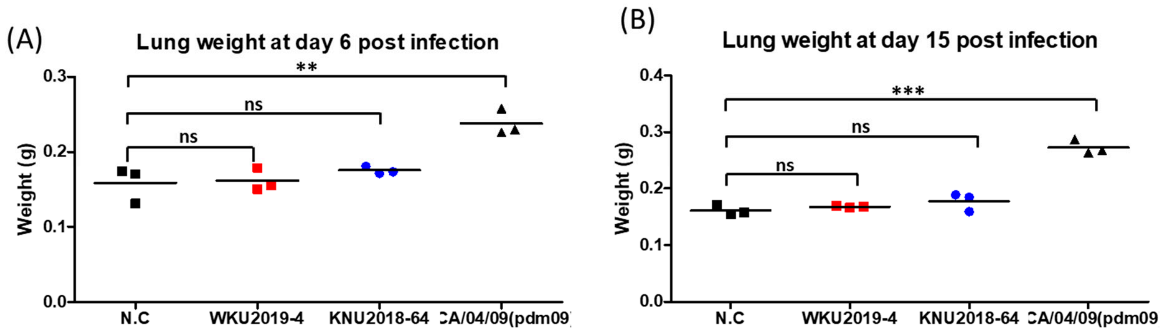

Figure S4. Lung weight at day 6 (A) and day 15 (B) post infection

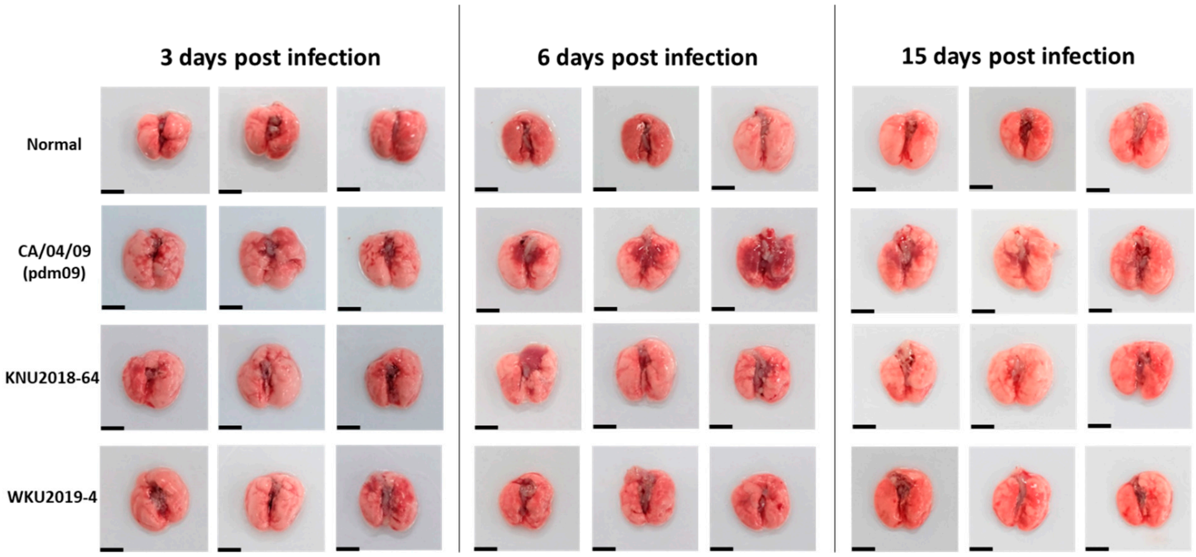

Figure S5. Lungs from infected mouse at day 3, day 6, day 15 post infection. Scale bar: 0.5 cm
